# Waste Coffee Silver Skin as a Natural Filler in PLA-Based Filaments for Fused Filament Fabrication (FFF) Printing

**DOI:** 10.3390/polym17131766

**Published:** 2025-06-26

**Authors:** Ana C. Machado, Ana F. Costa, Ângela R. Rodrigues, Pedro F. Moreira, Fernando M. Duarte, António J. Pontes

**Affiliations:** 1 IPC-Institute for Polymers and Composites, University of Minho, 4800-058 Guimarães, Portugal; gomesc.francisca@gmail.com (A.F.C.); angela.rodrigues@dep.uminho.pt (Â.R.R.); pedromoreira@dep.uminho.pt (P.F.M.); fduarte@dep.uminho.pt (F.M.D.); pontes@dep.uminho.pt (A.J.P.); 2 Done Lab-Advanced Manufacturing of Products and Tools, University of Minho, 4800-058 Guimarães, Portugal

**Keywords:** waste coffee silver skin, additive manufacturing, biobased materials, sustainable composite, circular manufacturing

## Abstract

In this research, novel biocomposite filaments were developed by incorporating coffee silver skin (CSS) waste into polylactic acid (PLA) for use in Fused Filament Fabrication (FFF) technology. CSS was blended with PLA at concentrations of 0, 5, 10, and 15 wt.% to address the waste disposal challenge and produce environmentally friendly composite biofilaments for FFF, supporting circular economic efforts. These filaments have the potential to be used in sustainable prototyping, functional parts, and consumer products. A comprehensive analysis was conducted to examine the effect of printing temperature on dimensional accuracy, melt flow index (MFI), and mechanical properties. Higher printing temperatures and increased CSS content led to larger dimensions due to increased material fluidity, as confirmed by MFI results, which increased from 3.5 g/10 min (0% CSS) to 5.8 g/10 min (15% CSS) at 180 °C, reaching 26.3 g/10 min at 220 °C. Tensile tests on 3D-printed specimens indicated an improvement in elastic modulus with increasing CSS content at lower temperatures (180 °C), rising from 1622 MPa (0% CSS) to 1952 MPa (15% CSS), representing about a 20% increase. However, at higher temperatures, the elastic modulus decreased, possibly due to the poor dispersion and agglomeration of filler particles. Tensile strength generally decreased with CSS addition, especially at higher loadings, while yield elongation remained low (~1.4–1.7%), indicating a more brittle material. The findings also revealed no significant thermal changes with increasing CSS content, and good printability was achieved for all compositions, which was characterized by good layer adhesion, the absence of warping, and the ease of extrusion.

## 1. Introduction

In response to growing concerns about environmental sustainability and waste management, industries increasingly focus on innovative solutions that minimize their ecological footprint. One promising approach is the circular economy, prioritizing resource reuse and waste minimization, which is gaining considerable attention in materials sciences and manufacturing [1,2]. The food industry, a significant contributor to organic waste generation, presents substantial opportunities for those innovations.

In recent years, the incorporation of natural fillers into polymer matrices has emerged as a sustainable strategy for developing new composite materials. Polylactic acid (PLA), a biodegradable polymer derived from renewable resources, has attracted significant attention due to its excellent mechanical and thermal qualities, eco-friendliness, biodegradability, and antibacterial qualities, making it a strong candidate material for sustainable biocomposites as well as 3D printing applications such as Fused Filament Fabrication (FFF) [3,4,5]. Adding plant fibers typically sourced from industrial or agro-industrial waste to PLA enables the development of composite filaments that not only reduce production costs, but also enhance the environmental degradability of the final products [6]. These biobased composites contribute to a more sustainable lifecycle for 3D-printed materials by promoting local resource use and improving the filament’s mechanical properties without compromising their biodegradability [7,8].

Ensuring consistent filament quality is essential for achieving mechanically reliable and dimensionally accurate prints in FFF processes. Inadequate control over filament dimensions and material homogeneity can lead to print defects such as clogging, poor interlayer bonding, and mechanical inconsistencies. Studies have shown that optimizing parameters such as barrel temperature, nozzle temperature, and motor speed significantly improved fiber distribution and dimensional stability in thermoplastic filaments [9].

Throughout the literature, it is possible to find many academic works related to the use of natural fillers to develop PLA biocomposites for several applications [10] and to produce filaments for FFF. Natural fillers include Bamboo [11], with wood fibers known as Timberfill [12], wood flour [13], olive wood [14], hemp fibers [15], soy hulls and soy protein [16], Acid-Bagasse [17], cork [18], Cocoa Bean Shell [19], and hemp hurd [20].

Coffee production, a global-scale industry, generates notable by-products such as coffee silver skin, a residue from the coffee-roasting process [21]. Although typically discarded or repurposed for low-value applications like fuel or fertilizer, CSS is now recognized as a potential renewable resource for advanced manufacturing applications [22,23]. Coffee silver skin, the thin fibrous layer that naturally detaches from coffee beans during the roasting process, represents the main solid by-product generated in coffee-roasting operations [24]. Coffee silver skin has recently emerged as a promising biobased resource for the development of sustainable polymer composites. Its inherently low moisture content, due to the roasting process, eliminates the need for energy-intensive drying [25]. As the primary by-product of industrial coffee roasting, CSS is abundantly available at processing facilities and can be readily collected in large quantities, making it a highly attractive and economically viable filler [26]. Furthermore, its naturally porous and fibrous structure appears to facilitate mechanical processing, particularly grinding, which is typically required before incorporation into polymer matrices [24,27]. These properties support its growing application in the formulation of sustainable biocomposites and highlight its potential as a circular economy solution for the coffee industry. The use of coffee waste, such as CSS and SCG to produce biocomposites, has also been studied by several authors [10,28,29,30,31]. Recent studies have shown that adding CSS into PLA not only reduces the environmental impact of plastic production, but also improves the mechanical and thermal properties of the resulting materials [10]. For example, CSS has been found to increase the elastic modulus of PLA composites while maintaining their printability and thermal stability, making it a promising natural filler for sustainable 3D printing [30].

Three-dimensional printing, particularly FFF, has emerged as a crucial technology for material recycling and waste repurposing thanks to its ability to produce complex geometries with minimal material loss. The use of biobased composites in FFF has facilitated the development of environmentally friendly filaments that reduce reliance on petroleum-derived materials while promoting the recycling of industrial by-products [32,33]. Studies comparing parts produced by 3D printing and injection molding show that injection molding generally yields superior mechanical properties, while 3D printing offers greater processing flexibility and design complexity. These insights are important for understanding the trade-offs and optimizing materials for specific applications [34,35].

This study aims to evaluate the feasibility of using coffee silver skin as a natural filler in PLA-based filaments for FFF printing. It examines how different CSS concentrations affect the extrusion behavior, mechanical properties, and dimensional accuracy of the printed components. Additionally, the performance of these 3D-printed parts is compared with that of specimens produced through injection molding to assess overall material performance and potential industrial applications. This research aims to promote sustainable manufacturing practices and highlights the importance of circular economy principles in modern production systems by incorporating waste materials into FFF printing filament fabrication.

## 2. Materials and Methods

### 2.1. Materials

Inzea^®^ F38, a PLA-based polymer, was purchased from Nurel S.A (Zaragoza, Spain). According to the manufacturer, it is a biobased and biodegradable PLA grade, suitable for the extrusion of 3D printing filaments, and contains over 70% renewable content. It has a density of 1.23 g/cm^3^ and a Melt Volume Flow Rate (190 °C, 2.16 kg) of 1.8 cc/10 min. Coffee silver skin was supplied by Delta Cafés. Before composite preparation, CSS was ground using a conventional blender and sieved through a 150 µm mesh using a Fritsch Analysette 3 sieve shaker. The sieved CSS particles had a maximum size of 150 µm, with no retained fraction above this threshold.

### 2.2. Production of Composites and Biofilaments for FFF

The composite materials were prepared with different concentrations of CSS (5 wt%, 10 wt%, and 15 wt%) using a laboratory modular Leistritz LSM 30.34 co-rotating twin-screw extruder. Inzea^®^ F38 without CSS (0 wt%) was also prepared as reference material. Before compounding, both Inzea^®^ F38 and CSS powders were oven-dried at 60 °C for 4 h to remove moisture. The screw speed was set to 100 r.p.m., with the temperature in the plasticizing unit ranging from 140 °C to 160 °C. The resulting composites were air-cooled at room temperature and then pelletized. The PLA/CSS granules were further dried (4 h at 60 °C in an oven) and used to produce filaments for FFF tensile specimen printing and for injection-molded tensile specimens for mechanical performance comparison. These granules were extruded into filaments using a 3Devo Composer 450 Filament Maker single-screw extruder (Utrecht, The Netherlands), whose conditions are presented in Table 1. The filament maker parameters were optimized through preliminary trial-and-error tests. The goal was to maintain stable extrusion flow and a consistent filament diameter and to avoid issues such as die swelling or nozzle clogging. Minor adjustments to the temperature profile were made to improve flowability while preventing the thermal degradation of both the polymer matrix and the CSS filler.

### 2.3. Sample Preparation for 3D Printing and Injection Molding

**Figure 1:**
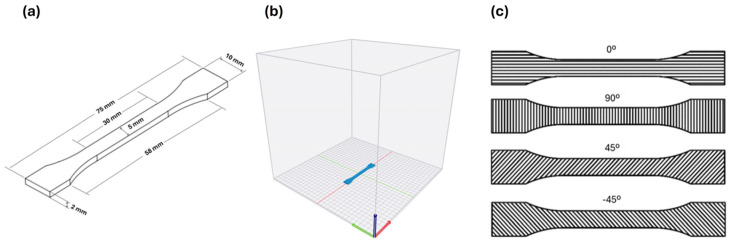
(**a**) Print orientation; (**b**) specimen dimensions according to the ISO 527-2 (2012) Type 1A standard; and (**c**) pattern orientation of the layers.

Standard dog-bone (type 1A) specimens, shown in Figure 1a, were printed from each PLA blend according to ISO 527-2 (2012) [36] using a Raise 3D Pro 2 printer with a flat print orientation, as depicted in Figure 1b [36]. The CAD model was created in SolidWorks 2024 (Dassault Systèmes S.A.,Vélizy-Villacoublay, France) and converted to a G-code file using Raise3D ideaMaker^®^ (version 4.3.3). All specimens were printed with a 100% infill, a layer height of 0.2 mm, and a pattern orientation of 0/90/45/−45 (Figure 1c) at a printing speed of 60 mm/s. A brass nozzle with a diameter of 0.4 mm was used, along with three different nozzle temperatures (180 °C, 200 °C, and 220 °C) and a platform temperature of 60 °C.

A Boy 22A injection molding machine produced tensile specimens (6 mm length × 3.9 mm width × 1.9 mm thickness). The processing temperatures ranged from 150 °C at the hopper to 190 °C at the nozzle, with the mold temperature at 25 °C. The injection molding process was carried out under an injection pressure of 80 MPa using a dosing volume of 22.8 cm^3^, a cooling time of 20 s, and a cycle time of 36 s.

### 2.4. Composites Characterization

#### 2.4.1. Melt Flow Index (MFI)

To evaluate the effect of CSS on the fluidity of the PLA matrix, the melt flow index of all prepared composites was measured using DAVENTEST equipment. The test was conducted with a 2.16 kg load at three different printing temperatures: 180 °C, 200 °C, and 220 °C. Three measurements were taken for each condition and the average MFI values along with their standard deviations were reported.

#### 2.4.2. Thermogravimetric Analysis (TGA)

Thermogravimetric analysis was performed on both the raw coffee silver skin and the PLA blends using a TGA Q500 (TA Instruments, New Castle, DE, USA). Ten milligrams of each sample were heated from 40 °C to 580 °C at 10 °C/min under a nitrogen atmosphere with a flow rate of 20 mL/min. The primary purpose of the TGA was to assess the thermal stability of CSS for melt extrusion processing with PLA. Additionally, the results provided a preliminary indication of the maximum temperature suitable for specimen printing.

### 2.5. Three-Dimensional Sample Characterization

#### 2.5.1. Measurement of Dimensional Accuracy

**Figure 2:**
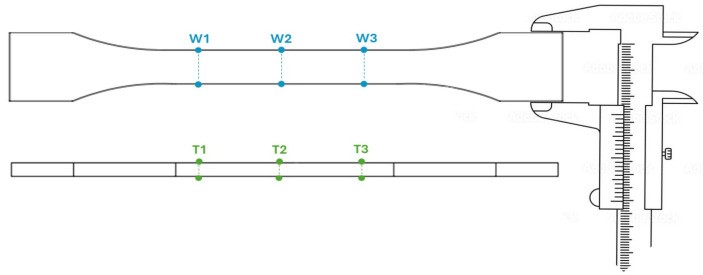
Measurement locations for width (blue markings) and thickness (green markings).

Ten specimens were produced and measured for each printing temperature. Each specimen had a gauge cross-sectional area of 5 mm × 2 mm (width × thickness), as shown in Figure 1b. Width and thickness measurements were taken at three different locations on each specimen, as illustrated in Figure 2, using a digital micrometer with an accuracy of ±0.01 mm. The average of these three measurements was compared to the CAD dimensions to evaluate dimensional accuracy. To assess the effect of printing temperature on the dimensional accuracy of the specimens, the measured dimensions were compared with the CAD dimension values. The absolute and dimensional errors were calculated using Equations (1) and (2), respectively. To quantify the variation, standard deviations were calculated for each set of measurements.

(1) $$Absolute\;Error\left(mm\right)=Measured\;Value-CAD\;Value$$

(2) $$Dimensional\;Error\;(\%)=\frac{\left(Measured\;Value-CAD\;Value\right)}{Measured\;Value}$$

#### 2.5.2. Tensile Testing

The mechanical properties of the PLA-CSS composites were evaluated using tensile tests using an Instron 5969 universal testing machine. These tests aimed to assess the effect of CSS content on the mechanical performance of PLA blends. Tensile specimens produced through both FFF printing and injection molding were tested at a crosshead speed of 10 mm/min using a 50 kN load cell at room temperature. A total of ten specimens from each blend were tested. The average values and standard deviations of elastic modulus, tensile strength, yield strength, and yield elongation were calculated.

#### 2.5.3. Optical Microscopy

The microstructure of the PLA-CSS composites was analyzed using a Leica DMS 1000 Stereo Microscope. Specimens printed at three different temperatures were bisected to examine their cross-sectional areas. Images were obtained using a Leica MTV-3 digital camera and then analyzed with Leica Application Suite software (version 4.13.0).

## 3. Results and Discussion

### 3.1. Filler Characterization

**Figure 3:**
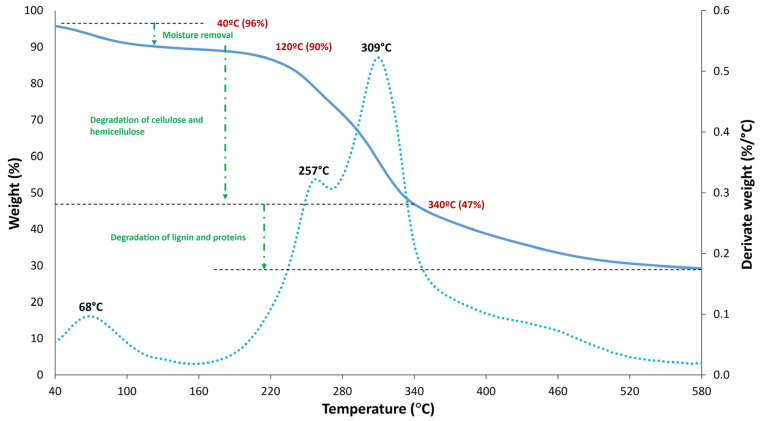
TGA and DTG thermographs of CSS.

Figure 3 presents the thermogravimetric analysis curves of CSS, where the bold line represents the weight loss and the dashed line represents the derivative weight loss. A three-step thermal degradation process is observed in CSS. The first weight loss, starting around 40 °C, is attributed to moisture evaporation, accounting for approximately 6% of the total weight loss. The second degradation stage shows a significant weight loss of about 43% due to the degradation of cellulose and hemicellulose components. The subsequent weight loss step, occurring above 340 °C, is attributed to the breakdown of lignin and proteins [37]. The derivative thermogram (DTG) peaks indicate the rate of temperature-dependent weight loss. When heating the CSS fillers, a deterioration peak around 68 °C corresponds to moisture removal. The thermal decomposition of hemicellulose and cellulose is represented by degradation peaks at 257 °C and 309 °C, respectively [38]. These results show that CSS has thermal stability like common natural fibers, with an onset decomposition temperature of about 210 ± 10 °C, indicating its suitability for processing with PLA as the processing temperature range of this polymer is well within this limit [39].

#### 3.1.1. Characterization of PLA-CSS Composites

1. Melt Flow Index

**Figure 4:**
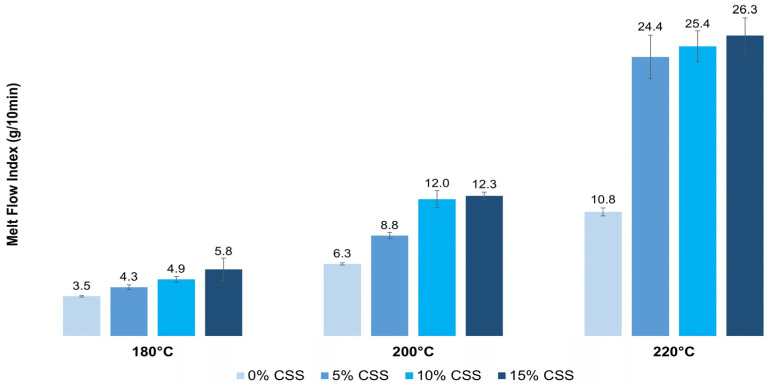
Melt flow index of PLA-CSS composites.

Figure 4 shows the melt flow analysis results for the prepared composite materials. Increasing the amount of CSS in the PLA matrix significantly enhances the material’s flowability at constant extrusion temperatures. This improvement is likely due to the presence of CSS fillers reducing polymer’s viscosity and facilitating flow. Similar trends have been observed in other biocomposites, where natural fibers, such as spent coffee grounds (SCG), improve melt flow by lowering the overall viscosity [30]. Additionally, the hydrophilic nature of the natural fibers, including CSS, may have caused the hydrolytic degradation of PLA during testing. Hydrolytic degradation involves the cleavage of ester bonds in the PLA backbone by water, leading to shorter polymer chains with a lower molecular weight [40]. Despite the drying process, CSS possibly retained some residual moisture, further contributing to the higher melt flow index. This phenomenon is well documented in studies on lignocellulosic fillers, where residual moisture promotes hydrolytic degradation during processing, even after drying [41,42].

The results also show that the MFI increases with higher temperatures, as expected for thermoplastic materials. The most significant change occurs between 200 °C and 220 °C, particularly in the composite with 5% CSS. This sharp increase in MFI at higher temperatures suggests that CSS enhances polymer chain mobility, possibly acting as a plasticizer. The plasticizing effect refers to the ability of certain substances to reduce intermolecular forces between polymer chains, thereby increasing chain flexibility and lowering viscosity. According to Kumar et al., proteins and lipids in CSS act as plasticizers in biopolymers and their presence contributes to a reduction in the storage modulus of PLA-based biocomposites, confirming this behavior [37].

These findings suggest that CSS content and processing temperature significantly influence the material’s flow properties. The melt flow rate of the PLA-CSS composites falls within the acceptable range for injection molding, as confirmed by the excellent processability seen during the injection molding test.

2. Thermogravimetric Analysis

**Figure 5:**
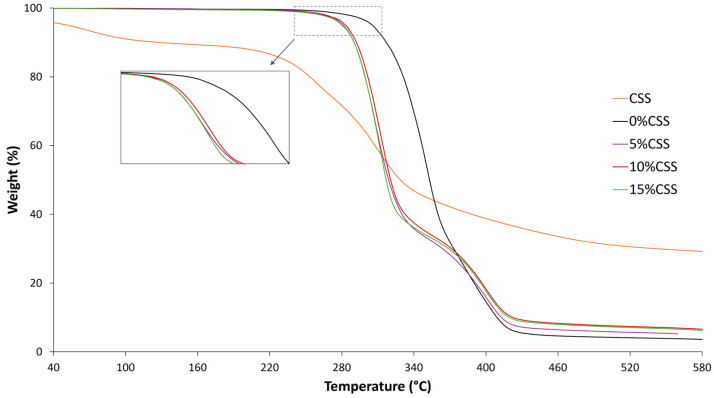
TGA thermographs of PLA-CSS composites.

Figure 5 illustrates the TGA curves of PLA-CSS composites comprising different filler loading from 0 to 15 wt%. All composites exhibit similar weight loss behavior as the neat polymer up to 220 °C. Beyond this temperature, the onset of degradation temperature slightly decreases with the addition of CSS filters. Similar observations have been reported in studies involving natural-fiber-reinforced PLA composites, where the thermal degradation characteristics are significantly influenced by the introduction of less thermally stable natural materials [7,43,44].

Moreover, while the incorporation of fillers reduces the thermal stability of the composites, it also leads to a decrease in the maximum rate of weight loss. This behavior is commonly observed, as the addition of natural fibers, which have a broader degradation window, tends to distribute weight loss over a wider temperature range [45]. The TGA results show that degradation starts above 220 °C, which led to the decision to limit the printing temperature to a maximum of 220 °C during the FFF printing process. This limitation is crucial to avoid the thermal degradation of the material, which could impair mechanical performance and print quality. Previous studies on PLA–natural fiber composites have also emphasized that maintaining printing temperatures below the onset degradation threshold is crucial to prevent nozzle clogging and ensure consistent extrusion [46].

#### 3.1.2. Challenges in Producing CSS-Filled PLA Filaments for FFF Printing

During the development of a fiber-reinforced thermoplastic composite for FFF printing, several challenges emerged. The production of fiber-based composite filaments, typically performed using a single screw, led to various issues, including the uneven dispersion of fillers within the polymer matrix, difficulties in temperature regulation, and the formation of voids during production [8]. To prevent nozzle clogging during printing, we determined that the CSS particle size needed to be minimized. A control filament was successfully produced without CSS, exhibiting a smooth surface. However, increasing the CSS content resulted in a rougher surface texture with visible clusters of CSS particles. The diameters of the produced filaments are summarized in Table 2.

**Figure 6:**
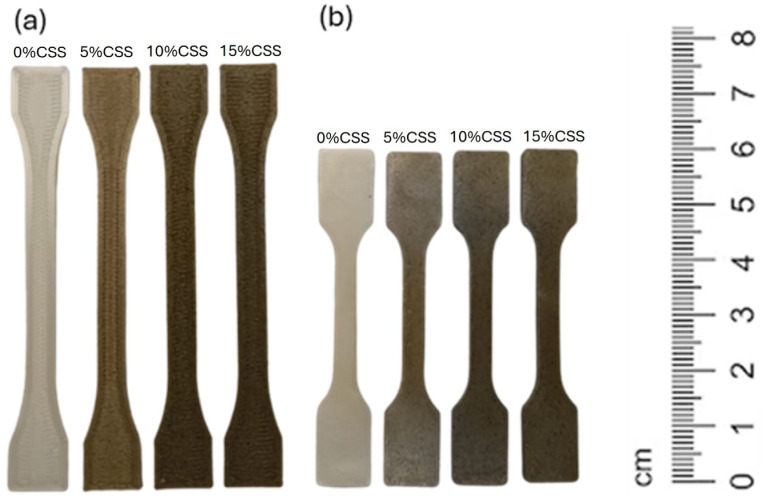
(**a**) FFF-printed specimens and (**b**) injection-molded specimens.

The same 3D model was printed using all filaments at three different nozzle temperatures. Figure 6a presents the 3D-printed specimens of all compositions produced at a nozzle temperature of 180 °C. The specimens produced through injection molding, using the same formulations and the temperature profile described in the methodology section, are shown in Figure 6b.

#### 3.1.3. Characterization of 3D Samples

1. Dimensional accuracy

Achieving high-quality FDM-printed parts that meet standards for shape, dimensional accuracy, stability, and repeatability are critical in the manufacturing process. Dimensional accuracy, a key factor in overall part quality, was evaluated for specimens printed with different temperatures and composite content, as shown in Table 3.

The results indicate that both concentration of CSS and printing temperature significantly influence the dimensional stability of the printed specimens. In general, higher CSS content and elevated temperatures lead to greater dimensional deviations from the nominal dimensions, particularly in thickness. Neat PLA specimens generally exhibited the lowest errors, while composites with15% CSS showed the highest deviation from the target dimensions. The observed deviations can also be attributed to changes in material viscosity at higher temperatures. As viscosity decreases, flowability improves, promoting lateral spreading and die-swell, which leads to dimensional deviations, a phenomenon also described by Turner et al. [47]. Moreover, the interaction between the PLA matrix and CSS fillers contributes to this behavior. As highlighted in recent studies, the thermal cycle experienced by thermoplastic polymers during FFF directly affects their microstructure and the physical properties of the printed parts. Higher printing temperatures promote the increased fluidity of the polymer matrix, compromising the stability of deposited layers and resulting in more pronounced dimensional deviations. This phenomenon is amplified with the inclusion of CSS. Natural fibers, such as CSS, are highly sensitive to temperature and moisture, and their polysaccharide composition can affect flow characteristics during printing. The literature suggests that natural fibers become increasingly sensitive to temperatures above 150 °C, disrupting the homogeneity of the polymer flow and leading to greater dimensional errors in composites with higher fiber content [48].

Although both dimensions exhibit increasing error with higher CSS concentrations and temperatures, the thickness measurements show a greater deviation than width. Errors in thickness reached over 10% in some cases, whereas the error in width generally remained below 5%. These errors may be partially attributed to temperature-induced stresses that arise during the layer-by-layer deposition process. Residual stresses accumulate due to differential cooling rates between layers, the polymer matrix, and the reinforcing fibers. The disparity in thermal expansion coefficients between the polymer and natural fibers, coupled with uneven cooling, possibly leads to dimensional inaccuracies [48].

These findings are consistent with previously discussed melt flow index data, which show that higher CSS content and printing temperatures increase flowability, though at the cost of dimensional accuracy. While CSS enhances melt flow, this benefit is offset by the resulting increase in dimensional inaccuracies. Therefore, optimizing both CSS content and printing temperature is essential to achieve the desired dimensional accuracy, especially for applications requiring precise geometries [49]. Furthermore, controlling thermal exposure time and the temperature profile is crucial for maintaining dimensional stability when working with natural-fiber-reinforced composites.

2. Bright field microscopy

**Figure 7:**
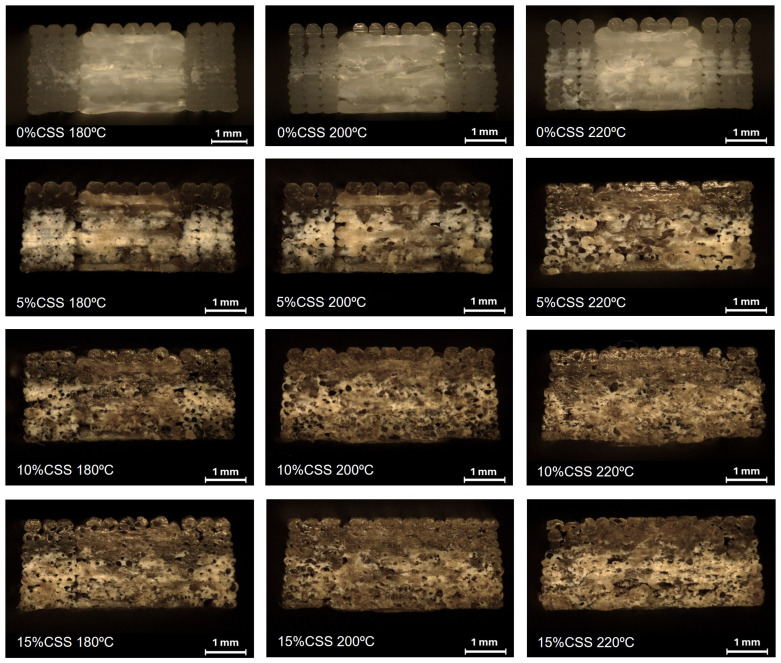
Micrographs of transversal section of printed specimens.

Figure 7 presents the cross-sectional images of the specimens printed at different temperatures for each composition.

The morphology of the PLA/CSS composites is significantly influenced by both the CSS concentration and printing temperature. The cross-sectional analysis highlights the key features affecting the microstructural characteristics, including particle distribution, layer adhesion, and the extent of porosity. These characteristics collectively determine the overall mechanical behavior of the material.

As the CSS content increases, particle agglomeration becomes more pronounced. At lower concentrations (up to 5% CSS), the distribution remains relatively uniform, whereas, at higher loadings (10% and 15% CSS), particle clustering becomes more prominent. The inclusion of fillers like CSS commonly modifies the rheological and thermal properties of the matrix, as well as its crystallization behavior, which can directly influence the printed composite’s microstructure [50]. While natural fillers like CSS can enhance mechanical interlocking at the matrix–filler interface—leading to better interfacial bonding—once agglomeration sets in, it creates stress concentrations that can locally weaken the composite. This observation aligns with studies on natural-fiber-reinforced composites, where moderate filler loadings tend to improve mechanical properties, but excessive filler leads to deterioration due to poor dispersion and particle clustering [51]. At higher CSS loadings (10% and 15% CSS), the cross-sectional images show increased surface roughness and irregularities. Although porosity is minimal in these composites, the rough texture is possibly caused by CSS particle agglomeration rather than void formation. Such agglomerates may act as micro-defects, weakening the composite’s mechanical properties by forming weak points in the matrix.

Regarding the influence of printing temperatures, the images suggest that higher temperatures improve layer fusion, as seen by the absence of distinct filament boundaries in specimens printed at 200 °C and 220 °C. According to Spoerk et al., higher temperatures are critical for achieving a balance between melt printability and raster bonding and avoiding feedstock degradation [52]. While higher temperatures generally enhance interlayer adhesion by increasing polymer chain mobility and diffusion, the presence of fillers like CSS and the formation of imperfect crystalline structures in the matrix may hinder polymer chain movement. This can reduce interface diffusion, potentially affecting the overall quality of the printed part. As seen in the MFI results, CSS likely acts as a plasticizer, increasing the flowability of the PLA matrix. This plasticization effect may mitigate void formation, especially at higher CSS contents, leading to denser printed parts. This observation is consistent with the literature, stating that the addition of plasticizers or flow-enhancing fillers typically reduces void formation and leads to denser printed parts [50]. Nevertheless, when the nozzle temperature becomes too high, the extruded filament may become excessively fluid, causing layer overlaps and resulting in suboptimal print quality [53].

3. Tensile properties of FFF-printed vs. injection-molded composites

**Figure 8:**
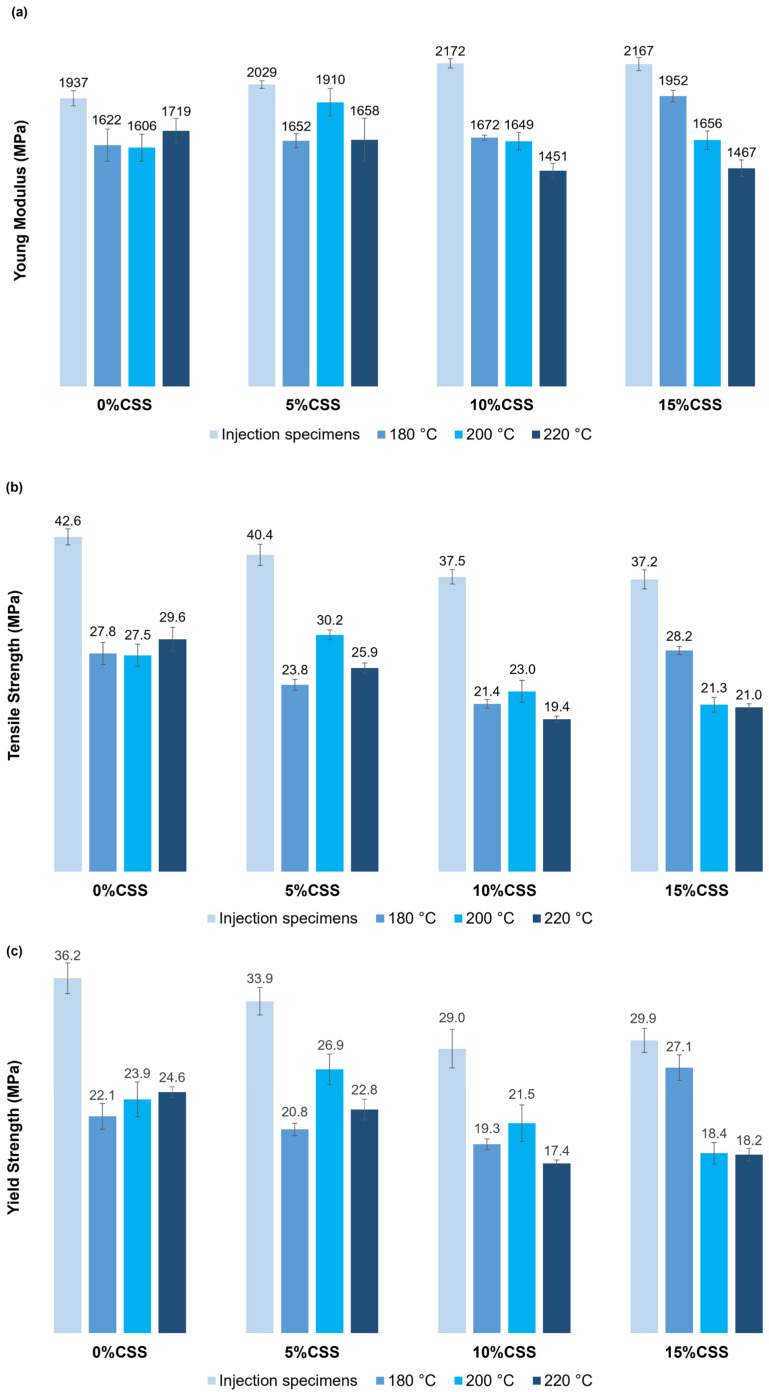

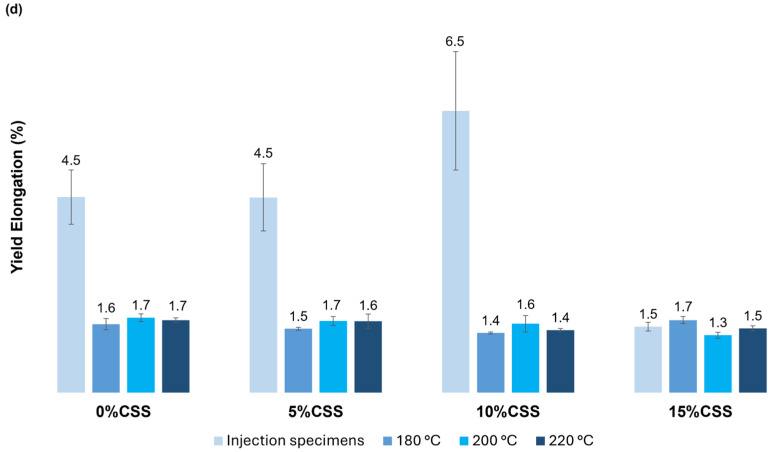
Mechanical properties of 3D-printed and injection-molded composites: (**a**) Young modulus; (**b**) tensile strength; (**c**) yield strength; and (**d**) yield elongation.

The overall average results of the mechanical properties are presented in Figure 8. The elastic modulus of injection-molded specimens increases with higher CSS content, primarily due to the incorporation of rigid lignocellulosic fillers that limit polymer chain mobility and increase material elastic modulus [37]. Injection-molded specimens consistently exhibit higher elastic modulus values than 3D-printed ones across all compositions and printing temperatures, largely because of weaker interlayer adhesion. Voids and weak interfaces in 3D printing can significantly reduce overall elastic modulus. In FFF-printed specimens, elastic modulus is influenced by printing and CSS content. At lower concentrations (up to 5% CSS), the effect of temperature on elastic modulus is minimal. Notably, the elastic modulus for 5% CSS at 200 °C approximates that of virgin injection-molded material, indicating an optimal balance between interlayer adhesion and filler dispersion. On the other hand, for higher CSS levels, elastic modulus decreases as the printing temperature rises. Cross-sectional images suggest increased particle agglomeration at these higher temperatures, creating microstructural defects that hinder load transfer and reduce elastic modulus. This increase in agglomeration can be attributed to the decreased viscosity of the molten composite at higher temperatures, which allows particles to migrate and cluster more easily. However, at lower CSS levels (5% CSS), better filler dispersion results in a more uniform stress distribution, leading to higher modulus values. Previous studies have reported that the incorporation of fillers modifies the polymer phase by forming an adsorbed polymer interphase at the filler surface. The degree of reinforcement achieved by adding fillers depends on several factors, including filler volume fraction, surface area, particle shape, adhesion quality between the filler and polymer, and the characteristics and thickness of the interphase formed between the two phases [54]. These factors justify the distinct behavior of the composite materials in this study.

For 3D-printed specimens without fillers (0% CSS), increasing the printing temperature slightly enhances tensile strength due to improved polymer chain diffusion and better interlayer bonding. However, upon the addition of CSS to the polymer matrix, a general decrease in tensile strength is observed. This reduction can be attributed to particle agglomeration and poor dispersion at higher CSS concentrations, leading to microstructural defects that impair load transfer. Prior research indicates that high printing temperatures can cause problems such as voids from overly fluid filament flow. These issues include bubbles, stringing, and inconsistent material deposition, all of which reduce tensile strength [46].

Yield strength also declines with increasing CSS content, mirroring the trends observed in tensile strength. Comparing injection-molded and 3D-printed specimens, the inherent weaknesses introduced by the FFF process become evident, especially at higher filler contents. Injection-molded specimens show significantly higher yield strength due to the more uniform distribution of CSS particles and the absence of layer-related defects that are typical in 3D-printed parts. Among printed specimens, those produced at 200 °C exhibit slightly higher yield strength than those printed at 180 °C. This improvement is likely due to better interlayer adhesion and reduced porosity, as seen in the cross-sectional images. However, CSS agglomeration limits the benefits of higher temperature on yield strength, particularly at 10% and 15% CSS content, where larger CSS clusters reduce the material’s load-bearing capacity.

Yield elongation remains relatively unaffected by printing temperature and CSS content. The addition of rigid CSS fillers further reduces ductility, resulting in a stiffer material.

## 4. Conclusions

This study successfully developed and evaluated PLA/CSS composites regarding their flow properties, dimensional accuracy, and mechanical performance. The findings demonstrate that incorporating coffee silver skin as a natural filler in PLA filaments for Fused Filament Fabrication (FFF) holds significant promise for enhancing sustainability and expanding potential applications in additive manufacturing technologies.

Key findings related to dimensional accuracy and mechanical behavior include the following:

- Increasing printing temperature and higher CSS content can compromise dimensional stability but enhance material fluidity.
- An optimal concentration of 5% CSS resulted in notable improvements in both mechanical properties and dimensional stability, indicating a balance between reinforcement and flowability.
- The 5% CSS composite exhibited consistently low dimensional errors in both thickness and width, indicating reduced sensitivity to temperature variations, making it suitable for applications where dimensional stability is crucial.
- Mechanical behavior was influenced by printing temperature, affecting interlayer adhesion and filler dispersion, particularly at higher CSS concentrations.

These results emphasize the importance of optimizing both printing temperature and filler content to achieve the best outcomes in terms of dimensional accuracy and mechanical performance.

Incorporating agro-industrial residues such as CSS into the production of FFF printing filaments represents a promising strategy to reduce environmental impact and promote circular economy practices in the additive manufacturing industry. Future studies should focus on optimizing processing conditions to minimize filler agglomeration and assessing the scalability of these materials for large-scale production.

## Figures and Tables

**Table 1:** Filament processing conditions.

Material	Temperature (°C)				Extrusion Rate (r.p.m.)
	Feed Zone	Compression Zone	Metering Zone	Die	
0% CSS	170	185	190	170	2.5
5% CSS	174	190	182	164	2.8
10% CSS	180	190	170	160	2.0
15% CSS	174	190	182	164	2.8

**Table 2:** Filament diameter (mm).

0% CSS	5% CSS	10% CSS	15% CSS
1.67 ± 0.10	1.69 ± 0.07	1.71 ± 0.07	1.72 ± 0.07

**Table 3:** Dimensions of FFF-printed specimens at different printing temperatures.

Temperature (°C)	Composite (% CSS)	Width (mm)				Thickness (mm)			
		5.00				2.00			
		Measured Value	Standard Deviation	Error ^(1)^		Measured Value	Standard Deviation	Error ^(1)^	
				mm	%			mm	%
180	0	5.01	0.06	0.01	0.1	2.11	0.01	0.11	5.6
	5	5.05	0.06	0.05	0.9	2.11	0.02	0.11	5.6
	10	5.08	0.01	0.08	1.5	2.10	0.02	0.10	4.8
	15	5.13	0.04	0.13	2.6	2.11	0.02	0.11	5.6
200	0	5.01	0.04	0.01	0.2	2.13	0. 00	0.13	6.4
	5	5.07	0.05	0.07	1.5	2.15	0. 03	0.15	7.7
	10	5.20	0.04	0.20	3.9	2.15	0. 02	0.15	7.4
	15	5.21	0.04	0.21	4.2	2.18	0. 02	0.18	8.9
220	0	5.05	0.02	0.05	1.0	2.14	0. 04	0.14	6.9
	5	5.07	0.06	0.07	1.5	2.16	0. 02	0.16	8.0
	10	5.23	0.04	0.23	4.6	2.21	0. 03	0.21	10.5
	15	5.25	0.07	0.25	5.1	2.21	0. 04	0.21	10.3

^(1)^ Absolute error in mm and dimensional error in %.

## Data Availability

The original contributions presented in this study are included in the article. Further inquiries can be directed to the corresponding author.
